# Age-Dependent Effects of A53T Alpha-Synuclein on Behavior and Dopaminergic Function

**DOI:** 10.1371/journal.pone.0060378

**Published:** 2013-04-01

**Authors:** Adam W. Oaks, Maya Frankfurt, David I. Finkelstein, Anita Sidhu

**Affiliations:** 1 Laboratory of Molecular Neurochemistry, Department of Biochemistry and Molecular & Cellular Biology, Georgetown University Medical Center, Washington, D.C., United States of America; 2 Department of Science Education, Hofstra North Shore-LIJ School of Medicine, Hempstead, New York, United States of America; 3 Florey Institute of Neuroscience and Mental Health, University of Melbourne, Victoria, Australia; University of Nebraska Medical Center, United States of America

## Abstract

Expression of A53T mutant human alpha-synuclein under the mouse prion promoter is among the most successful transgenic models of Parkinson's disease. Accumulation of A53T alpha-synuclein causes adult mice to develop severe motor impairment resulting in early death at 8–12 months of age. In younger, pre-symptomatic animals, altered motor activity and anxiety-like behaviors have also been reported. These behavioral changes, which precede severe neuropathology, may stem from non-pathological functions of alpha-synuclein, including modulation of monoamine neurotransmission. Our analysis over the adult life-span of motor activity, anxiety-like, and depressive-like behaviors identifies perturbations both before and after the onset of disease. Young A53T mice had increased distribution of the dopamine transporter (DAT) to the membrane that was associated with increased striatal re-uptake function. DAT function decreased with aging, and was associated with neurochemical alterations that included increased expression of beta-synuclein and gamma synuclein. Prior to normalization of dopamine uptake, transient activation of Tau kinases and hyperphosphorylation of Tau in the striatum were also observed. Aged A53T mice had reduced neuron counts in the substantia nigra pars compacta, yet striatal medium spiny neuron dendritic spine density was largely maintained. These findings highlight the involvement of the synuclein family of proteins and phosphorylation of Tau in the response to dopaminergic dysfunction of the nigrostriatal pathway.

## Introduction

Although not diagnosed clinically until the onset of motor impairment, Parkinson's disease (PD) is frequently associated with non-motor symptoms, including autonomic dysfunction, sleep abnormalities, and neuropsychiatric disorders [1]. Neuropsychiatric co-morbidities can significantly affect quality of life, and do not always respond to therapies targeting PD motor symptoms [2]. Nonetheless, the development of models used to study PD in pre-clinical research settings has been focused primarily on generating a robust motor phenotype that recapitulates features of the human disorder, especially α-synuclein (α-Syn) accumulation [3]. Accumulation of α-Syn in intraneuronal structures called Lewy bodies is a neuropathological hallmark of PD, and one approach to modeling the disease has been to create transgenic mice that over-express the A53T mutant of α-Syn, which causes an autosomal dominant form of PD in humans [4]. Mouse genomes have been modified to over-express wild-type α-Syn of human origin [5], as well as the PD-linked α-Syn mutants A53T [6]–[9], A30P [10], E46K [11], and even combinations thereof [12]. The A53T α-Syn mouse model successfully recapitulates many important features of synucleinopathy, including age-dependent neurodegeneration [3]. Accumulation and aggregation of A53T α-Syn in the highest-expressing transgenic lines is associated with the development of severe motor impairment resulting in early death at 8–12 months of age [6]–[8]. As pre-clinical α-Syn models of PD that may be used to test new therapies, it is critical to determine whether the neuropsychiatric phenotypes of α-Syn transgenic mice accurately reflect the human disorder. Both the dopamine transporter (DAT) and the norepinephrine transporter (NET), which are subject to modulation by the Syn proteins in cellular trafficking models [13], are linked to these neuropsychiatric symptoms, and are important drug targets in the treatment of depression, anxiety, and related mood disorders [14]. Recent work in mouse models of synucleinopathy has begun to examine non-motor aspects, including behaviors related to the neuropsychiatric symptoms of PD, but a comprehensive analysis remains incomplete (see detailed reviews in [15], [16]).

In pre-symptomatic A53T mice, altered locomotor activity and loss of anxiety-like behaviors have been reported, though the effect of A53T α-Syn varies significantly between the transgenic lines examined [17]–[19]. Furthermore, the presence of depressive-like behavior in these animals, to our knowledge, has not been analyzed, despite the fact that depression is a frequent co-morbidity of PD [20]. Behavioral changes that precede severe A53T-related neuropathology may stem from disruption of the normal functions of α-Syn and the other Syn family members, β-Syn and γ-Syn, including modulation of monoamine neurotransmission [13]. For example, we have observed recently that all three forms of the Syn proteins can modulate DAT and are co-distributed with DAT in the mouse brain (unpublished data), and earlier work showed that NET is modulated by the Syn family of proteins as well [21]. In particular, our prior work suggests that defective modulation of DAT by A53T α-Syn may contribute to behavioral and neurochemical changes in the A53T mouse model of PD [22], [23].

The present analysis in homozygous A53T α-Syn mice over much of the adult life-span of locomotor activity, anxiety-like, and depressive-like behaviors identifies perturbations both before and after the onset of motor impairment. In addition to behavioral alterations, we show that over-expression of A53T α-Syn had age-dependent effects on re-uptake of DA, and that functional modulation of DAT in these animals was correlated with age-dependent changes in the striatal accumulation of α-Syn, β-Syn, and γ-Syn. Furthermore, we report age-dependent accumulation of phosphorylated Tau and activation of Tau kinases, which we and others have shown co-occur in several PD models [24]-[29]. We also found that, despite a significant loss of substantia nigra pars compacta neurons, striatal synaptic density was maintained. Together, our findings suggest a mechanism by which defective modulation of monoamine neurotransmission by mutant A53T α-Syn could be involved in the clinical manifestations of synucleinopathy.

## Materials and Methods

### Ethics Statement

All studies with animals were approved by the Georgetown University Institutional Animal Care and Use Committee (Protocol 10-076).

### Materials

Primary antibodies used on immunoblots in this study are listed in Tables 1 and 2. Radio-labeled dopamine ([^3^H]-DA, NET131, 24 Ci/mmol) and norepinephrine ([^3^H]-NE, NET377, 13.8 Ci/mmol) were purchased from Perkin Elmer (Waltham, MA). All other reagents, except where indicated, were purchased from Sigma-Aldrich (St. Louis, MO).

**Table 1:** Synuclein, transporter, and other antibodies.

Target^a^	Source	Product	Host^b^	Dilutions^c^	
α-Syn	BD Transduction	610787	Ms	1∶2000	
α-Syn (h)	Invitrogen	18-0215	Ms	1∶500	
β-Syn	Novus Biologicals	NB100-79903	Rbt	1∶1000	
γ-Syn	Abcam	ab55424	Rbt	1∶2000	
DAT	Millipore	MAB369	Rt	1∶2000	
NET (m)	MAb Technologies	NET05-2	Ms	1∶1000	
β-Actin	Santa Cruz	sc-1616	Gt	1∶1000	
Cadherin	Abcam	ab6528	Ms	1∶2000	
GAPDH	Cell Signaling	2118	Rbt	1∶3000	
TH	Millipore	ab152	Rbt	1∶300	

a Protein recognized by antibody. Species specific antibodies indicated in parentheses (h, human; m, mouse). ^b^Antisera raised in mouse (Ms), goat (Gt), rabbit (Rbt), or rat (Rt). ^c^Antibody dilutions applied for immunoblots or immunohistochemistry.

**Table 2:** Tau and Tau kinase antibodies.

Target^a^	Source	Product Number	Host^b^	Dilutions^c^
Tau-5	Millipore	MAB361	Ms	1∶1000
p-T181-Tau	Anaspec	54960-025	Rbt	1∶500
p-S199-Tau	Invitrogen	44734G	Rbt	1∶500
CP-13 Tau	P. Davies^d^	-	Ms	1∶500
p-T212-Tau	Invitrogen	44740G	Rbt	1∶500
p-S214-Tau	Invitrogen	44742G	Rbt	1∶500
p-T217-Tau	Invitrogen	44744	Rbt	1∶500
p-T231-Tau	Invitrogen	44746G	Rbt	1∶500
p-S262-Tau	Invitrogen	44-750G	Rbt	1∶1000
PHF-1 Tau	P. Davies^d^	-	Ms	1∶500
p-S422-Tau	Abcam	ab4862	Rbt	1∶500
Akt	Cell Signaling	2966	Ms	1∶500
p-S473-Akt	Cell Signaling	4058	Rbt	1∶500
Cdk5	Santa Cruz	sc-6247	Ms	1∶500
ERK	BD Transduction	610124	Ms	1∶500
p-Y204-ERK	Santa Cruz	sc-7383	Ms	1∶1000
GSK-3β	BD Transduction	610202	Ms	1∶1000
p-Y216-GSK-3β	BD Transduction	612313	Ms	1∶1000
JNK	Cell Signaling	9252	Rbt	1∶500
p-T183/Y185-JNK	Cell Signaling	9255	Ms	1∶500
p38	Santa Cruz	sc-7972	Ms	1∶500
p-p38	Santa Cruz	sc-166182	Ms	1∶500

a Protein or phosphorylated epitope recognized by antibody. ^b^Antisera raised in mouse (Ms) or rabbit (Rbt). ^c^Antibody dilutions applied for immunoblots. ^d^Antibody provided as a generous gift by Peter Davies.

### Animals

Previously described mice homozygous for a mutant human A53T-α-Syn transgene driven by the prion promoter (A53T) were acquired from the National Institute of Aging (NIA) Mutant Mouse Aging Colony [8]. Age-matched wild-type controls (WT) were acquired from the NIA Aged Rodent Colony. Animals were imported to Georgetown University at 2-12 months of age from NIA colonies directly prior to studies and allowed to acclimate for several days before beginning behavioral testing.

### Animal behavior

All animals were subjected to an identical battery of behavior tests over a three week period, with at least 48 hours between each test. Testing was conducted in a quiet (50–55 dB ambient noise), dedicated room. Tests are described below in the order in which they were conducted according to previously described methods [19], [21], [30]-[32].

#### Open field test

Locomotor activity and anxiety-like behavior were assessed using the open field test (OFT) as described previously [19]. The animals were placed in the center of the activity chamber (40×40 cm with clear 35 cm high walls) equipped with a camera above to record activity. Testing lasted for 10 min per animal. The exploratory behavior for each animal was analyzed automatically using the ANY-maze video tracking system (Stoelting, Wood Dale, IL). The analysis included distinguishing activity within a center zone of the open field to assess thigmotaxis. The chamber was divided into 16 equal squares, with the center zone defined as the middle four squares (equal to 25% of the total area of the box). Line breaks were determined by the passage of the animal's center from one square into a neighboring square.

#### Rotarod

The rotarod apparatus (IITC Life Sciences, Wood Hills, CA) was used to measure balance and motor coordination [19]. During the training period, mice were allowed to explore the cylinder of the rotarod (71 cm long with diameter 3.2 cm) for 2 min without rotation. Each mouse was then oriented with its head opposite the direction of rotation and the drum was slowly accelerated to a speed of 4–40 rpm over a period of 300 sec. The latency to fall off the rotarod within this time period was recorded (up to 300 sec). Mice received nine trials (three per day for three consecutive days), and the mean latency to fall off the rotarod was calculated.

#### Forced swim test

A previously described forced swim test (FST) protocol [21] was adapted for mice using a glass cylinder (15 cm diameter) filled to a depth of 13 cm with room temperature water. Mice were pre-tested for five min 24 hours prior to the test period. During the five min test period, animals were placed again into the testing cylinder, and their behavior was recorded with a horizontally-mounted camera. Videos of each animal were assessed by a blinded observer, and scored for the behavior present (swimming or immobility) in each five s bin. Data are presented as number of bins spent immobile.

#### Elevated plus maze

The elevated plus-maze (EPM) test was performed as described previously [19]. The elevated plus-maze apparatus (Stoelting) was elevated 40 cm above the floor and consisted of four arms measuring 35 cm in length and 5 cm in width. Two of the arms (closed) were enclosed with opaque 15-cm high walls, and two arms had no walls (open). Mice were placed with their head in middle of the center zone at the junction of the open and closed arms. During the 5-min test session the exploratory behavior of each animal was analyzed using the ANY-maze video tracking system (Stoelting). Analyses included the time spent and number of entries into the center zone, open arms, and closed arms. Zone entries were scored automatically by ANY-maze when the center of the animal passed into the respected zones. The maze was sponged clean between trials. Animals that fell from the maze were immediately replaced in the starting position; tracking errors were identified by review of the recorded videos and corrected manually.

#### Wire hang test

The wire hang test (WHT) was used to assess grip strength and endurance as described previously [30]–[32]. Mice were placed on a wire cage top in an identical orientation and induced to grip the wires by gently waving the cage top. The cage top was then inverted and suspended at a height of 40 cm over an open cage filled with bedding and excess nesting materials to prevent injury from falling. Mice received one training session, and were then placed on the wire hang apparatus twice per day for three consecutive days for a total of six trials. Movement was recorded by a horizontally mounted camera, and trials were ended automatically by ANY-maze (Stoelting) when the animal fell from the suspended cage top, with a maximum duration of 300 s. Some 12 month-old A53T mice were unable to hang from the wire cage; these animals were given a score of zero s for all trials and euthanized for tissue collection as below.

### Tissue collection

Following completion of behavioral testing, animals were euthanized by saline perfusion under anesthesia (intraperitoneal 10 mL/kg equithesin) or cervical dislocation. Saline perfused animals were further perfused with 4% paraformaldehyde in preparation for immunohistochemical analysis (see below). For biochemical analyses, brain tissue was removed and processed for synaptosomal uptake assays or protein extraction. To isolate the striatum and hippocampus, a razor blade was first used to slice coronally through the brain at approximately Bregma −0.5. A wedge-shaped section of non-striatal tissue was then removed from the rostral portion of the brain, which was then halved sagittally. Each half striatum was then dissected away from surrounding cortical tissues. The caudal portion of the brain was then halved sagittally and diagonal slices were cut at −60° from the horizontal plane, starting at the left and right ventricles and extending through the cortex. The cortical tissue was peeled back to reveal the surface of each half of the hippocampus, which was then removed. Striatal and hippocampal tissues from each animal were individually flash frozen in liquid nitrogen or processed immediately for synaptosomal uptake assays.

### Synaptosome preparation

Synaptosomes were prepared from each animal essentially as described with slight modifications [21], [33]. Dissected striatal and hippocampal tissues were placed in 2 mL ice cold synaptosome isolation buffer (0.32 M sucrose, 4 mM HEPES, 1 mm EDTA; pH 7.4) with Complete Mini Protease Inhibitor Cocktail (Roche, Indianapolis, IN). Tissue was homogenized in glass–Teflon homogenizers on ice, transferred to polypropylene centrifuge tubes (Beckman, Brea, CA), and diluted to 6 mL with isolation buffer. Brain homogenates were centrifuged at 1000×g for 10 min at 4°C. The supernatant was collected and centrifuged at 12,500×g for 15 min at 4°C, producing the synaptosome pellet. The synaptosome pellet was resuspended in 1 mL of a modified Kreb's HEPES buffer (120 mM NaCl, 7.5 mM HEPES, 5.4 mM KCl, 5 mM Tris HCl, 5 mM glucose, 1.2 mM CaCl_2_, 1.2 mM MgSO_4_, 1.0 mM ascorbic acid, 1.0 µM pargyline HCl; pH 7.4). The protein concentration was determined by Lowry assay (Biorad), the synaptosomes were diluted to 80–100 µg/mL (striatal synaptosomes) or 250–300 µg/mL (hippocampal synaptosomes) in modified Kreb's HEPES buffer, and used for uptake experiments.

### Synaptosomal uptake assay

Uptake assays were performed on freshly prepared synaptosomes as described previously [21], [33]. Tissues from each animal were assayed independently (no tissues were pooled), and each data point was taken as the average of samples assayed in triplicate. For each sample, 300 µL of the synaptosomal preparation was placed in a glass assay tube and incubated at 37°C for 10 min with shaking, followed by the addition of [^3^H]-DA at a final concentration 30 nM or [^3^H]-NE at a final concentration of 150 nM. Uptake proceeded for 10 min and was terminated by plunging samples into an ice water bath. Filtration was immediately performed on a 24-sample Brandel Cell Harvester (Gaithersburg, MD) and samples were collected on GF/C Whatman filters that were presoaked in 0.1% polyethyleneimine. Filtered samples were washed three times with 5 mL of ice cold assay buffer. Filters were collected, scintillation cocktail was added, and counts per minute determined using a Beckman Liquid Scintillation counter. Nonspecific uptake of [^3^H]-DA and [^3^H]-NE was determined with the addition of 100 µM indatraline HCl or 1 µM desipramine HCl, respectively, 5 min prior to initiation of uptake. Additional non-specific control assays were performed in parallel for [3H]-NE uptake with the addition of 300 nM nisoxetine HCl or incubation at 4°C.

### Brain tissue protein extraction

Frozen intact brain tissues stored at −80° C were thawed briefly then homogenized in ice cold homogenization buffer (10 mM Tris HCl, 100 mM NaCl, 1 mM EDTA, 1 mM EGTA, 250 mM sucrose, pH 7.4) with Complete Mini Protease Inhibitor Cocktail (Roche) and Halt Phosphatase Inhibitor Cocktail (Thermo Scientific, Rockford, IL). Homogenate protein concentration was determined by Lowry assay (Biorad), adjusted, and aliquots were frozen at −80°C until further extraction by various methods.

#### Cholate extraction

Previously described methods were used to prepare total lysates [27]. Briefly, to solubilize protein, sodium cholate (20% in water, wt/vol) was added to a final concentration of 1% (vol/vol) and samples were incubated for one hour with rotation before being processed for immunoblots.

#### Preparation of Triton X-100 soluble and insoluble fractions

The aggregation state of α-Syn was analyzed based on its differential solubility in 1% Triton X-100, as described previously [24], [34]. Briefly, tissue homogenates were extracted in solubilization buffer (20 mM Tris-HCl, 50 mM NaCl, 1% Triton X-100, pH 7.4) with Complete Mini Protease Inhibitor Cocktail (Roche) and Halt Phosphatase Inhibitor Cocktail (Thermo Scientific). Lysates were incubated for 30 min on ice, followed by centrifugation at 15,000×g for 60 min at 4°C and supernatant was collected as the Triton X-100- soluble fraction. The Triton X-100-insoluble pellets were re-dissolved in the same volume of lysis buffer containing 2% SDS [34] then analyzed by immunoblot.

#### Synaptosomal plasma membrane extraction

A plasma membrane fraction was isolated from synaptosomes as described previously [35], [36]. Briefly, synaptosomes were prepared as described above then lysed by hypo-osmotic shock and homogenization in ice cold water with protease inhibitors. HEPES was immediately added to a final concentration of 4 mM and synaptosomes were incubated 30 min then centrifuged at 30000 RCF for 20 min to yield the synaptosomal membrane pellet. Pellets were extracted with the addition of nonidet P-40 (1%), sodium deoxycholate (0.5%), and SDS (0.1%) before analysis by immunoblot.

### Immunoblot analysis

Samples were combined 1∶1 with Laemmli Sample Buffer (Bio-Rad) containing 5% β-mercaptoethanol. Samples were boiled for 5 min, cooled to room temperature, loaded on 10–20% Tris-HCl Criterion gels (Bio-Rad), separated by electrophoresis, and electroblotted onto polyvinylidene fluoride membranes. After blocking with 20 mM Tris-buffered saline, pH 7.6 containing 0.1% Tween 20 and 5% (wt/vol) blotting grade blocker non-fat dry milk (Bio-Rad) for 1 hour at room temperature, primary antibodies were applied overnight at 4°C in blocking buffer at manufacturer recommended dilutions. Washed immunoblots were incubated for 2 hours at room temperature with HRP-conjugated secondary antibodies (1∶3000; Santa Cruz) and proteins were revealed by enhanced chemiluminescence (Perkin Elmer, Waltham, MA). Immunoblot films were digitized with an Epson Perfection V700 Photo Scanner and quantified using ImageJ.

### Immunohistochemistry and stereological counting of substantia nigra pars compacta neurons

The 4% paraformaldehyde perfused brains were transferred to 4% paraformaldehyde solution overnight, then transferred to 30% sucrose in PBS and stored at 4°C for approximately one week. Brains were serially sectioned using a cryostat (Leica) at a thickness of 30 µm, sampling 1 in 3 sections through the substantia nigra pars compacta (SNpc). A complete series of SNpc sections was used for TH immunohistochemistry (1∶300, Millipore, USA) as described previously [37] and counter stained for Nissl substance with neutral red (Nissl, Grale Scientific, Victoria, Australia). The total number of DA neurons in the SNpc was estimated using a fractionator sampling design [38]–[40]. Counts were made at regular predetermined intervals (x = 140 µm, y = 140 µm). Systematic samples of the area occupied by the nuclei were made from a random starting point. An unbiased counting frame of known area (45 µm×35 µm) was superimposed on the image of the tissue sections using stereology software (MBF, Stereo Investigator) utilizing a 63×objective lens (Leica, N.A.1.36). Experimenters were blinded to the treatments of each of the groups.

### Analysis of striatal synaptic spine density

Brains were removed and right hemisphere was placed in solutions provided in the Rapid GolgiStain Kit (FD NeuroTechnologies, Ellicott City, MD) to perform Golgi impregnation according to the manufacturer's instructions. Primary dendrites from medium spiny neurons (MSN) of the striatum were analyzed blindly. Six cells per animal that were well impregnated, clearly distinguishable from adjacent cells, and with continuous, unbroken dendrites were chosen for analysis as described previously [41]. Spines were counted with an oil objective (100×) using a Nikon Eclipse E400 microscope and the entire dendritic length visible was measured using Spot Advanced Microscopy software (SPOT Imaging Solutions, Diagnostic Instruments, Inc., Sterling Heights, MI). Spine density was calculated by dividing the number of spines by the length of the dendrite and data was expressed as number of spines per 10 µm of dendrite.

### Statistical analysis

Results are expressed as mean ± standard error of the mean (SEM) unless stated otherwise. Behavior tests and immunoblots comparing the genotypes at each age are analyzed using 2-way ANOVA, with Bonferroni post-tests performed between A53T and WT at each age (2, 4, 8, and 12 months). Immunoblot analysis involving age-dependent changes are analyzed by 1-way ANOVA within each genotype. Unless noted, all other comparisons are made using un-paired two-tailed t-tests. Statistical significance was accepted at P<0.05 and is denoted with a single asterisk (*). Additional statistical distinctions are made at P<0.01 (**) and P<0.001 (***).

## Results

### Reduced locomotion and age-dependent loss of motor performance in A53T mice

**Figure 1:**
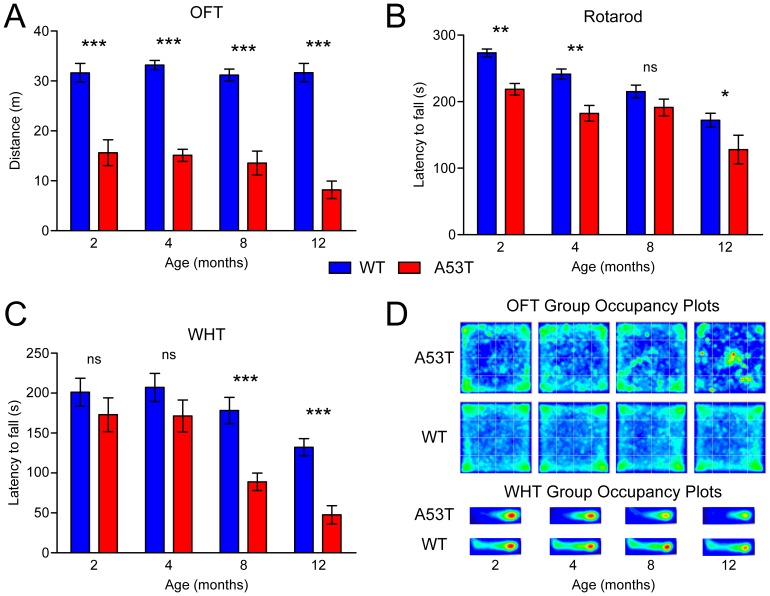
Motor activity and function. Open field (OFT), rotarod, and wire hang tests (WHT) were performed on WT and A53T mice at 2–12 months of age (n = 14–18 per group) to analyze motor activity and function. (A) Total distance traveled on the OFT was measured over a ten min period by automated video tracking using ANY-maze software. (B) Latency to fall on the rotarod was recorded by a blinded observer in nine trials performed over three days. (C) Latency to fall from the inverted grid on the WHT was recorded by automated video tracking using ANY-maze software. (D) Heat map occupancy plots from the OFT and WHT were averaged from recorded behavior of all animals in each group. Results are presented as mean ± SEM and were analyzed by two-way ANOVA with Bonferroni post-hoc tests comparing each A53T group to age-matched controls (**p<0.01; ***p<0.001).

To assess locomotor activity and motor performance, homozygous A53T mice were compared to WT mice at 2, 4, 8, and 12 months on several behavior tests (see Materials and methods) including the open field test (OFT), elevated plus maze (EPM), rotarod, and wire hang test (WHT). At all ages, A53T mice showed significantly reduced locomotor activity compared to WT, as quantified by line breaks on the OFT (Fig. S1A) and distance traveled on both the OFT (Fig. 1A) and the EPM (Fig. S1B). Rotarod latency to fall was also reduced in A53T mice at 2, 4, and 12 months (but not 8) compared to WT (Fig. 1B). Latency to fall on the rotarod declined with aging at a similar rate in both genotypes, and suggested relatively minor impairment of coordination in A53T mice (Fig. S1C). The reduced activity level of A53T mice was also evidenced by occupancy plots from the OFT and on the inverted grid of the WHT (Fig. 1D). Though baseline motor activity was reduced at all ages, the strength and endurance measured by the WHT were not affected until advanced ages. Latency to fall on the WHT was significantly reduced only in 8 and 12 month-old animals (Fig. 1C). Together, these data indicated a reduced level of locomotor activity in homozygous A53T mice that was apparent at all ages, while pronounced loss of function was observed only in 8 and 12 month-old mice. The development of strength and endurance impairments at 8 months corresponds well with the previously reported timing of severe motor impairment in several high-expressing A53T mouse lines [6]–[8]. Symptomatic mice were largely or completely unable to perform on the WHT, with latency of less than 20 s. Though the observed motor phenotype was largely consistent with previous reports, there was no indication that homozygous A53T mice in this study were hyperactive at any age; indeed, A53T mice were consistently less active than age-matched WT animals.

### Age-dependent loss of anxiety-like and depressive-like behavior in A53T mice

**Figure 2:**
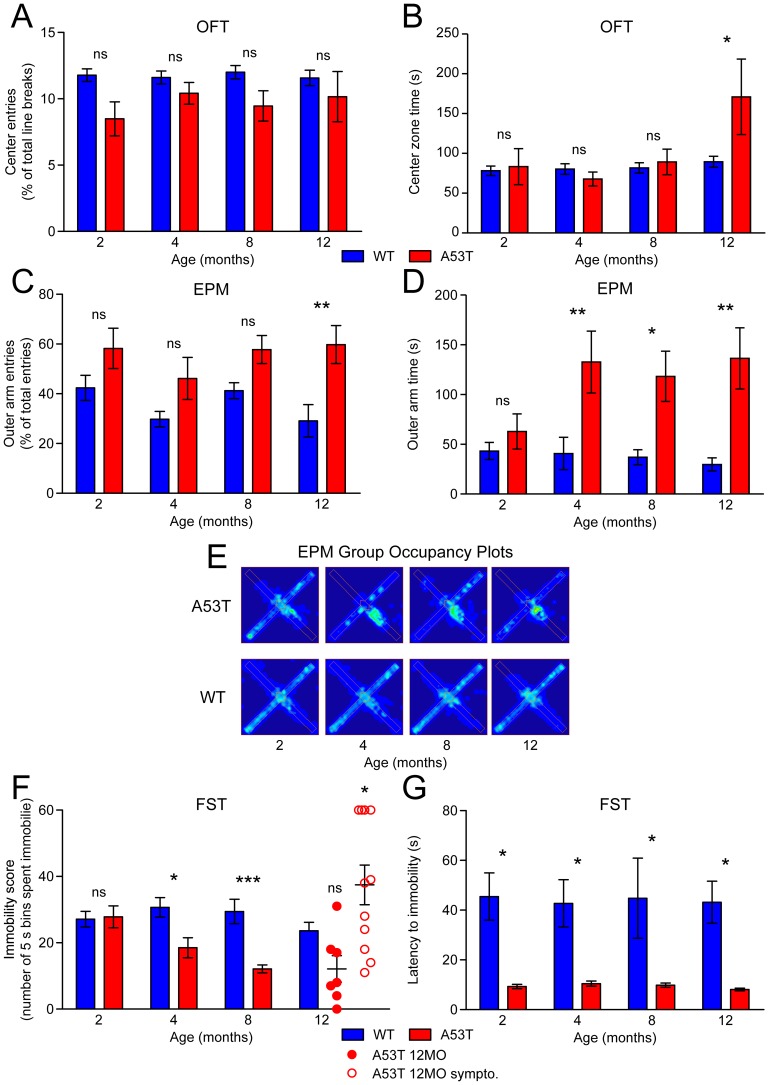
Anxiety-like and depressive-like behavior. Anxiety-like behavior was analyzed by the OFT and elevated plus maze (EPM) on WT and A53T mice at 2-12 months of age (n = 14–18 per group). (A) Center zone entries and (B) time on the OFT, and (C) open arm entries and (D) time on the EPM were measured by automated video tracking using ANY-maze software. (E) Heat map occupancy plots from the EPM were averaged from recorded behavior of all animals in each group. Depressive-like behavior was determined from the (F) immobility score and (G) latency to first immobility on the forced swim test (FST). Behavior was monitored from video recordings by a blinded observer counting the number of 5 s bins each animal spent in an immobile posture. 12 month-old A53T immobility score data is split into non-symptomatic (A53T 12MO; filled red circles) and symptomatic (A53T 12MO sympto.; open red circles). Results are presented as mean ± SEM and were analyzed by two-way ANOVA with Bonferroni post-hoc tests comparing each A53T group to age-matched controls (*p<0.05; **p<0.01; ***p<0.001).

Behavior on the OFT and EPM was also monitored for classical anxiety-like phenotypes, including thigmotaxis and aversion for elevated or open spaces. We have shown previously that hemizygous A53T mice have decreased anxiety-like behavior with aging [19], and others have found reductions in anxiety related phenotypes in 2 month-old homozygous mice [42], though older animals were not studied. Here we examined the homozygous A53T mice at 2, 4, 8, and 12 months to assess the development of anxiety-like behavior across the adult life span and through the progressive loss of motor function. On the OFT the center zone entry count was significantly reduced at all ages in A53T mice (Fig. S2A). This corresponded with a reduced overall activity level (Fig. 1A), and when taken as a fraction of total entries, OFT center zone entries were unchanged (Fig. 2A). Time spent in the OFT center zone, however, was significantly elevated in A53T mice at 12 months (Fig. 2B), producing a clearly observable shift to the center in the OFT occupancy plot (Fig. 1D). On the EPM, the fraction of outer arm entries was increased at 12 months (Fig. 2C), while time spent in the EPM outer arms was significantly increased at 4, 8, and 12 months (Fig. 2D). Total outer arm entry counts were unchanged at 2 and 12 months, with significant decreases in 4 and 8 month-old A53T mice (Fig. S2B). Nonetheless, the lack of aversion for the EPM open arms was apparent in the occupancy plots, which show that older A53T mice spent a significant amount of the time on the outer arm at the EPM junction (Fig. 2E). Thus, A53T mice demonstrated a consistent loss of anxiety-like behavior with aging, failing at later ages to manifest stereotypical behaviors including thigmotaxis and avoidance of exposure.

A53T mice also showed reduced depressive-like behavior with aging as indexed by the FST (see Methods). At 2 months, A53T mice scored similarly to WT animals, spending on average about half of the testing period in an immobile posture (Fig. 2F). In older A53T mice, however, FST immobility scores declined, indicating that the animals spent more of the test period swimming or attempting to escape. Immobility was significantly reduced in 4 and 8 month-old A53T mice (Fig. 2F). At 12 months the FST behavior of A53T mice diverged depending on the deterioration of motor function. In symptomatic 12 month-old A53T mice (WHT latency<20 s) the trend toward decreased depressive like behavior was reversed, as these animals had significantly elevated immobility scores compared to age-matched WT (Fig. 2F), while average immobility scores for non-symptomatic 12 month-old A53T animals stayed low. Surprisingly, while the total immobility score decreased with aging, the latency to first immobility was significantly decreased in A53T mice at all ages (Fig. 2G). A53T mice of all ages tended to freeze immediately upon entering the water, though these animals on average generated reduced immobility scores across the test period. Together, OFT, EPM, and FST data indicate an overall reduction in anxiety-like and depressive-like behavior in aging A53T mice.

### Monoamine transporter distribution and re-uptake capacity

**Figure 3:**
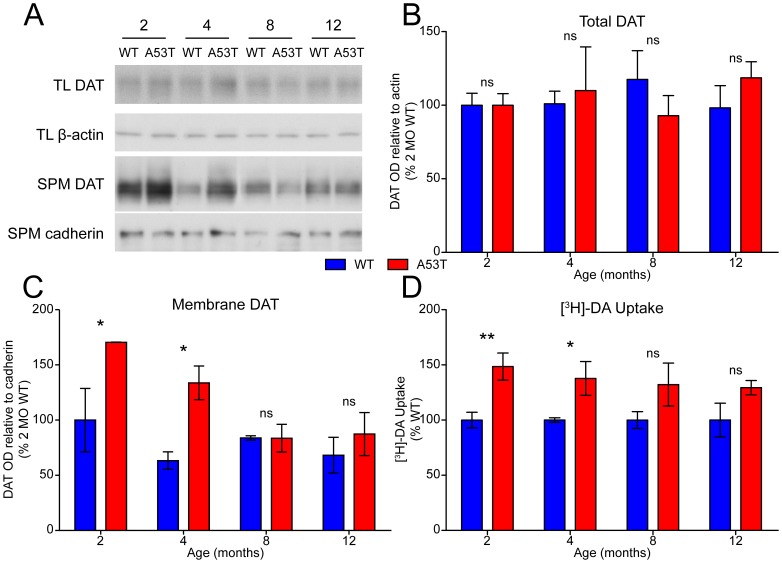
DAT distribution and function. Expression and distribution of DAT was analyzed by immunoblot on protein from (A) total lysates (TL) or synaptosomal plasma membrane fractions (SPM). Actin or cadherin expression, respectively, were analyzed as loading controls. Representative blot images from each genotype at each age are presented with approximate molecular mass of nearest protein ladder bands indicated (M_r_). Band optical density (OD) of (B) TL and (C) SPM DAT relative to loading controls is presented as percent of two month-old WT (mean ± SEM) and was analyzed by two-way ANOVA with Bonferroni post-hoc tests comparing each A53T group to age-matched controls (*p<0.05). (D) Uptake of [^3^H]-DA into striatal synaptosomes isolated from WT and A53T mice at 2–12 months of age was measured in triplicate from six animals per group and is presented as percent of age-matched WT control (mean ± SEM). Non-specific uptake was determined in the presence of 100 µM indatraline HCl and has been subtracted. Comparisons between WT and A53T α-Syn at each age were made by t-test (*p<0.05, **p<0.01).

To probe the possible involvement of DA and NE re-uptake in the phenotype of A53T mice, expression, distribution, and function of DAT and NET were examined. In the striatum, expression of DAT does not differ significantly between genotypes, nor is there an effect of aging (Fig. 3A–3B). Immunoblot analysis of a synaptosomal plasma membrane fraction (SPM) showed, however, that DAT levels were significantly elevated at the cell surface in the striatum of 2 and 4 month-old A53T mice, but not older ages (Fig. 3A, 3C). Similarly, striatal synaptosomes also had an increased capacity for uptake of [^3^H]-DA at 2 and 4 months in A53T mice, while uptake of [^3^H]-DA is comparable to WT in older animals (Fig. 3D). Unlike striatal DAT, expression and SPM distribution of NET in the hippocampus were unchanged at all ages (Fig. S3A-S3C). Though uptake of [^3^H]-NE into hippocampal synaptosomes was decreased slightly in A53T mice at some ages (Fig. S3D) there was no consistent effect of aging. It remains unclear how Syn proteins affect NE dynamics in the mouse brain, with some effects on NE levels in A53T mice [43] and re-uptake of NE in A30P mice [44] reported previously. Membrane distribution and re-uptake function of DAT, however, were consistently elevated in younger A53T animals, but normalized at older ages (Fig. 3D).

### Aged-dependent accumulation of synuclein proteins in the striatum

**Figure 4:**
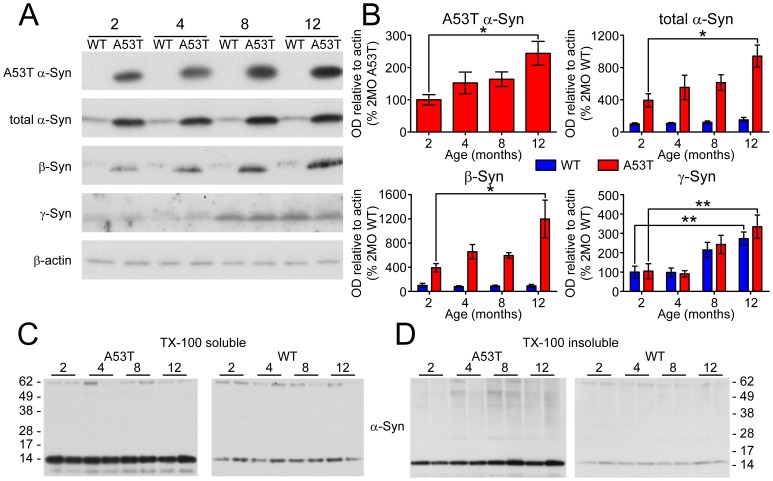
Synuclein accumulation and aggregation in the striatum. (A) Expression of Syn proteins was analyzed by immunoblot on striatal total lysates with actin expression analyzed as a loading control. (B) Band optical density (OD) relative to actin is presented as percent of two month-old A53T mice (A53T human α-Syn) or two month-old WT (total α-Syn and endogenous Syn proteins) and was analyzed by two-way ANOVA with Bonferroni post-hoc tests comparing each A53T group to age-matched controls (mean ± SEM; *p<0.05; **p<0.01). (C) Solubility of α-Syn was analyzed by immunoblot on striatal homogenate extracted with 1% TX-100 and centrifuged at 15,000 RCF for 60 min. Insoluble pellets were further extracted with the addition of 2% SDS and fractions were analyzed in parallel by immunoblot. Representative blot images from each genotype at each age are presented with approximate molecular mass of nearest protein ladder bands indicated (M_r_).

To determine the progression of biochemical changes associated with the behavioral and neurochemical phenotype of homozygous A53T mice, Syn protein expression was analyzed at 2, 4, 8, and 12 months of age. A53T α-Syn is expressed only in A53T mice (Fig. 4A), producing a 4–8 fold increase in the total α-Syn load in the striatum (Fig. 4B). Striatal A53T α-Syn expression appears to increase with age (Fig. 4A), and is significantly elevated in 12 month-old A53T mice compared to 2 month-old A53T mice (244+/−83%, P<0.01; Fig. 4B). β-Syn expression is elevated by 4–12 fold in A53T mice (Fig. 4B), and appears to increase with age in these animals (Fig. 4A). Indeed, expression of β-Syn is significantly increased in 12 month-old A53T mice compared to 2 month-old A53T mice (305+/−178%, P<0.05; Fig. 4B). Interestingly, no differences in γ-Syn expression between A53T and WT mice are present, though striatal γ-Syn levels appear to increase with age in both animals (Fig. 4A). Expression of γ-Syn in 12 month-old animals of both genotypes is significantly elevated compared to corresponding 2 month-old mice (WT, 273+/−77%, P<0.01; A53T, 320+/−128%; Fig. 4B). Regression analysis of immunoblot data confirms that expression of synuclein family members α-Syn and β-Syn increased with age in A53T mice, while γ-Syn accumulated with age in both A53T and WT mice (Table 3). There is evidence that accumulating α-Syn in the striatum is in part composed of synuclein oligomers. While high molecular weight α-Syn is absent in Triton X-100 soluble fractions (Fig. 4C), 4, 8, and 12 month-old A53T have detectible oligomeric species (Fig. 4D). These species are absent at all ages and in both fractions from WT mice.

**Table 3:** Regression analysis of synuclein expression.

Genotype	Synuclein protein	Slope^a^	R^2^	p-value^b^
A53T	human A53T α-Syn	22.16	0.4148	**0.0022**
	total α-Syn	17.80	0.2835	**0.0156**
	β-Syn	29.97	0.3355	**0.0074**
	γ-Syn	40.20	0.4971	**0.0005**
WT	human A53T α-Syn	NA^c^	NA^c^	NA^c^
	total α-Syn	8.256	0.1786	0.0634
	β-Syn	-0.6978	0.0010	0.8922
	γ-Syn	31.77	0.5121	**0.0004**

a Slope calculated from linear regression of immunoblot optical density of the indicated Syns measured at 2-12 months, where 2-month-old mean of respective genotypes is set to 100% and all other values are expressed relative to 2-month-old level (also measured as percents, see Fig. 4). Slope units are percent change per month of age. ^b^Statistical test for non-zero slopes for each regression analysis. Statistically significant p-values (<0.05) are bolded. ^c^Human A53T α-Syn is not present in WT mice.

Over-expression of both α-Syn and β-Syn was readily apparent in the hippocampus (Fig. S4A) but the effect of aging was absent, with roughly the same increase in hippocampal synuclein load present in A53T mice at all ages, and no change in γ-Syn expression (Fig. S4B). Oligomeric α-Syn was not detected in the hippocampus (Fig. S4C-S4D), which is consistent with previous reports showing the absence of hippocampal α-Syn inclusions in aged A53T mice [8]. Taken together, increased expression of β-Syn and γ-Syn and the shift in solubility of A53T α-Syn constitute a change in the relative bioavailability of the various Syn proteins in the striatum.

### Age-dependent activation of Tau kinases and accumulation p-Tau

**Figure 5:**
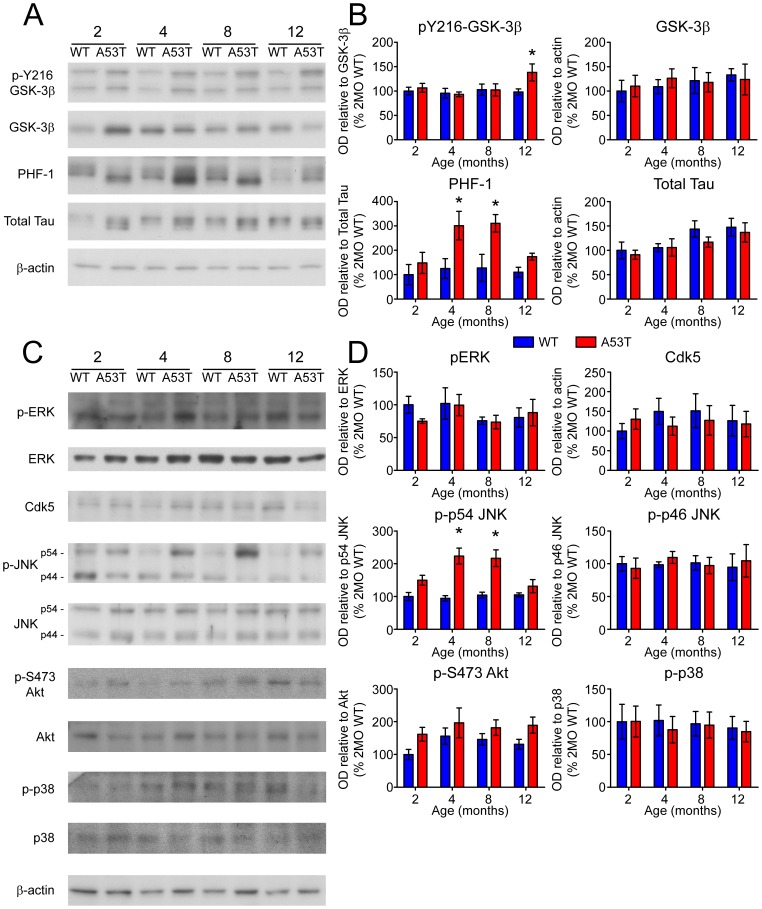
Accumulation of PHF-1 Tau and Tau kinase activation. (A) Phosphorylation of Tau protein at the PHF-1 epitope, expression of Tau kinases, and phosphorylation of kinases at activating sites was analyzed by immunoblot. (B) Band optical density (OD) relative to appropriate loading controls is presented as percent of 2 month-old WT (mean ± SEM) and was analyzed by two-way ANOVA with Bonferroni post-hoc tests comparing each A53T group to age-matched controls (*p<0.05).

Increased uptake of DA (see Fig. 3) implies an excess of intracellular DA in the striatum, which is a known promoter of oxidative stress and has been linked to neurodegenerative processes [45], [46]. Dopaminergic toxins that produce significant oxidative stress can simultaneously induce hyper-phosphorylation of Tau (p-Tau), a marker of neurodegenerative pathology, through an α-Syn dependent mechanism [47]–[49]. Indeed, we and others have shown that p-Tau, hyper-phosphorylated at numerous sites, accumulates in the striatum and other brain regions in mouse models of PD, including α-Syn transgenic mice [24], [25], [28] as well as mice treated with PD-linked agricultural toxin paraquat [27]. As a measure of neuropathological progression in A53T mice, accumulation of PHF-1 Tau (phosphorylated at Ser396/Ser404), an indicator of Tau hyper-phosphorylation, was initially analyzed by immunoblot in the striatum (Fig. 5A) and hippocampus (Fig. S5A) of 2, 4, 8, and 12 month-old animals. In accordance with previous work, PHF-1 Tau was significantly elevated in the striatum of 4 (201±59%, P<0.05) and 8 month-old A53T animals (161±57%, P<0.05, Fig. 5B). An activating phosphorylation at Y216 of GSK-3β (p-GSK-3β), a Tau kinase, was also increased in these animals, although at a later age (38±17%, P<0.05, Fig. 5B). Accumulation of p-Tau and increased phosphorylation of GSK-3β were not observed in the hippocampus (Fig. S5A-S5B). Thus, in the striatum where DAT trafficking is disrupted and Syn protein accumulation occurs, p-Tau formation and activation of GSK-3β also increase. Where age-dependent Syn protein accumulation and dopaminergic dysfunction are absent, PHF-1 Tau and p-GSK-3β increases are likewise not observed. This is consistent with our prior observation that hyper-phosphorylation of Tau in PD brains is largely restricted to striatal tissues [50], where dopaminergic innervation is the highest [51].

**Figure 6:**
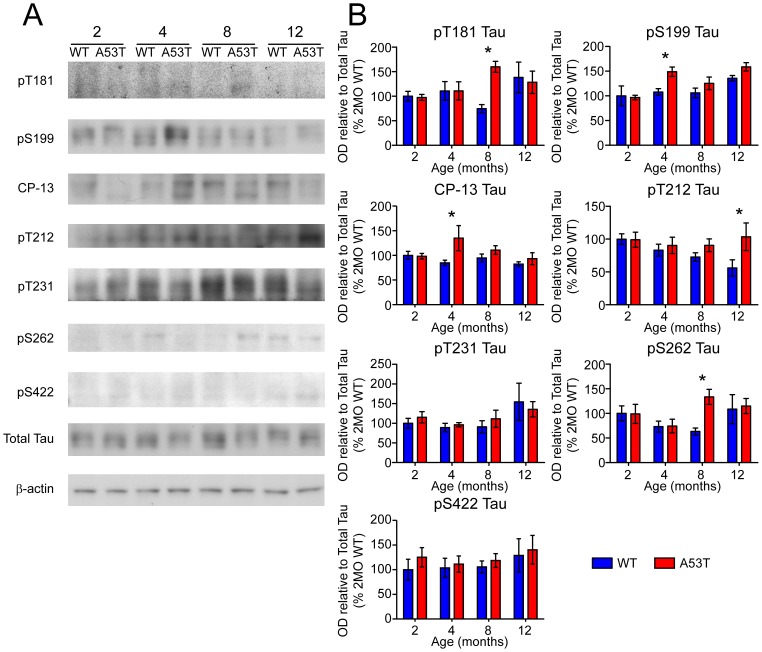
Screen for additional pJNK-Tau sites in striatum. (A) Phosphorylation of Tau protein at epitopes subject to phosphorylation by JNK was analyzed by immunoblot. (B) Band optical density (OD) from phosphorylation-specific probes relative to total Tau expression is presented as percent of 2 month-old WT (mean ± SEM) and was analyzed by two-way ANOVA with Bonferroni post-hoc tests comparing each A53T group to age-matched controls (*p<0.05).

Early PHF-1 Tau accumulation in the striatum occurred prior to significant activation of Tau kinase GSK-3β. In order to identify other kinases potentially responsible for increased levels of Tau phosphorylation, a panel of known Tau kinases was screened by immunoblot (Fig. 5C) for expression and phosphorylation levels (Fig. 5D). Among the additional putative Tau kinases probed, only JNK (stress activated/Jun-amino-terminal kinase) had a consistent pattern of increased phosphorylation (Fig. 5C), as an activating phosphorylation of the p54 isoform of JNK at T183 and Y185 (p-JNK) was significantly elevated in A53T mice at 4 and 8 months (Fig. 5D). JNK has been shown previously to phosphorylate many sites of full length Tau [52], and is activated in the A30P α-Syn mouse model of synucleinopathy [28]. Therefore, antibodies specific to these phosphorylation sites of Tau were used to probe for accumulation of p-Tau (Fig. 6A). Several additional sites that are known JNK substrates were increased at different time points, including pS181, pS199, CP-13, pT212, and pS262 (Fig. 6B).

**Figure 7:**
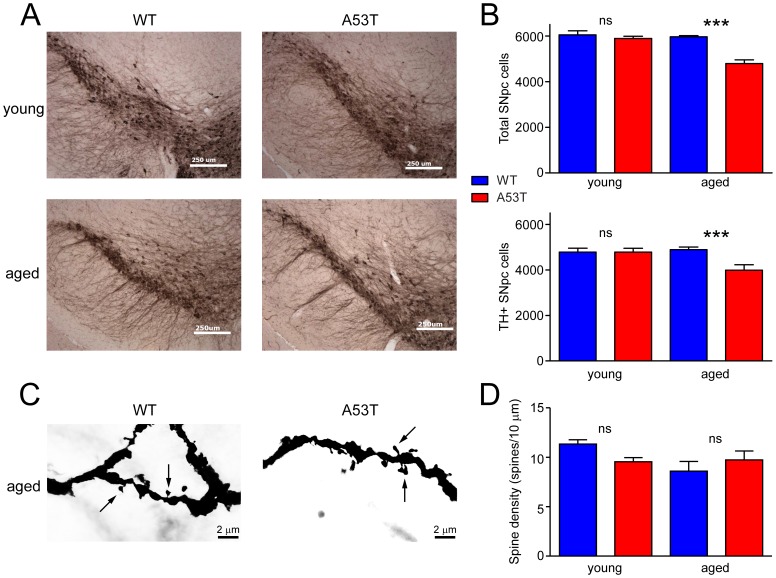
Assessment of nigrostriatal neuropathology. (A) Representative images show anatomical extent of TH-expressing neurons in young (two months) or aged (12 months) WT and A53T mice. (B) Abundance of TH-expressing (TH+) and total neurons in the substantia nigra pas compacta (SNpc) was analyzed by quantitative stereology in young (2–4 months) and aged (8–12 months) mice (n = 5–10 per group). Data are presented as mean±SEM and were analyzed by two-way ANOVA with Bonferroni post-hoc tests comparing each A53T group to age-matched controls (*p<0.05; ***p<0.001). (C) Representative images show dendritic spine morphology in striatal medium spiny neurons from 10 month-old WT and A53T mice. (D) Dendritic spine density was quantified from six spines per animal in young (two months) and aged (10 months) mice (n = 3–6 animals per group). Data are presented as mean ± SEM and were analyzed by t-tests comparing each A53T group to age-matched controls; no significant differences detected.

The integrity of the dopaminergic nigrostriatal pathway was also analyzed. In agreement with our previous work [24], a significant loss of total and TH-expressing neurons was detected in the substantia nigra pars compacta (SNpc) of aged A53T mice (Fig. 7A–7B). Surprisingly, despite the loss of SNpc neurons, total DAT expression was not affected over the life span of A53T mice (Fig. 3A–3B), consistent with compensatory sprouting of DA terminals [38]. Likewise, striatal post-synaptic integrity appeared to have been largely maintained, as dendritic spine counts on medium spiny neurons (MSN) in this brain region are unchanged, even in aged animals (Fig. 7C–7D). Together, these data indicate partial sparing of striatal connectivity despite neuronal losses in the SNpc and severe synucleinopathy known to exist in other brain regions [6]–[8].

## Discussion

Our documentation of motor impairment, behavioral perturbations, altered neurochemical function, and the development of synucleinopathy and tauopathy over the adult lifespan of A53T mice provides a novel view of these processes in a well-studied PD model. Our analysis has clarified the relationship between the motor phenotype of these mice and the apparent loss of anxiety-like behavior, suggesting that the latter is largely a consequence of or at least concomitant with reduced locomotion. In addition, the results of our study illustrate two phenomena critical to our understanding of how the Syn proteins function in both normal and pathological circumstances.

First, we provide evidence that a change in the local concentration of α-Syn, β-Syn, and γ-Syn can be associated with functional modulation of DAT in the mouse brain. In cellular models of DAT trafficking, A53T α-Syn binds poorly to DAT and fails to modulate DAT trafficking to the cell surface [22], [53]. Similarly, DA uptake and DAT distribution to striatal membranes are increased in young A53T mice where bioavailability of abundant A53T α-Syn is highest. This supports the view that the loss of α-Syn modulation of DAT through over-expression of trafficking incompetent A53T α-Syn results in increased localization of DAT to the cell surface. Uptake of DA through DAT was normalized in older animals where bioavailability of A53T α-Syn was reduced and expression of β-Syn and γ-Syn was increased. Indeed, we have observed recently that all three wild-type Syn proteins have a similar capacity to modulate DAT distribution (unpublished data). The normalization of DA uptake with aging may therefore relate to a shift in modulation of DAT from α-Syn to the other Syn proteins β-Syn and γ-Syn. The progressive removal of trafficking-incompetent A53T α-Syn and replacement with β-Syn and γ-Syn may also indicate a compensatory response involved in the restoration of normal DAT trafficking. The effects on NET of A53T α-Syn have not been examined previously, and though α-Syn-NET and α-Syn-DAT interactions are mediated by the same NAC region [22], [23], these studies produced no conclusive result regarding the modulation of NET by A53T α-Syn.

Second, we propose that mis-trafficking of DAT in the presence of excess A53T α-Syn contributes to biochemical alterations in the striatum, including increased levels of hyper-phosphorylated Tau (p-Tau). We have shown that over-expression of trafficking-incompetent A53T α-Syn in young mice leads to increased DAT distribution to the cell surface and elevated intracellular DA exposure. Subsequent to these changes in DA uptake we observe increased activation of JNK and Tau hyperphosphorylation at several JNK-targeted epitopes. Importantly, phosphorylation at the PHF-1 epitope and other p-Tau sites in the striatum appeared to resolve after 8 months, which correlated with the restoration of DA uptake to normal levels. Tau phosphorylation returned to normal levels at all but one site in 12 month-old A53T mice, indicating that p-Tau accumulation is transient and not likely to be contributing to alterations in DAT trafficking or function. Thus, despite significant loss of SNpc neurons and severe synucleinopathy known to occur throughout the brain stem and spinal cord [8], [54], striatal structural integrity appears to have been largely preserved. The transient nature of increased Tau phosphorylation and lack of striatal pathology suggests that observed changes in endogenous Syn protein expression at later ages could have restored Syn-dependent DAT trafficking and therefore be involved in alleviation of DA-induced stress kinase signaling.

Modulation of DAT trafficking through a direct interaction with α-Syn was initially described over ten years ago [55], and subsequent work has identified several potential mechanisms that could contribute to trafficking of DAT by α-Syn [56] as well as the other Syn family proteins β-Syn and γ-Syn (unpublished data). While modeling of Syn-DAT trafficking in mammalian cell culture has been informative, prior studies have been unsuccessful in detecting Syn modulation of DAT, with most reporting negative findings [57]–[59]. These results therefore constitute the first evidence for a dynamic relationship between Syn protein concentration and DAT function in the mouse brain. Of particular interest is the correlation between excess DA uptake, stress kinase activation, increased Tau phosphorylation, and changes in Syn expression. It is currently unknown whether these events are coordinated, and the mechanisms involved have not been identified. A proposed compensatory response involving increased expression of Syn family members β-Syn and γ-Syn is consistent with evidence for redundancy between the three Syn proteins that is regulated at the transcriptional level [60], and indicates the need for further investigation into the mechanisms that control Syn gene expression.

Here, we have attempted to overlay changes in the integrity and function of the nigrostriatal pathway with age-dependent changes in anxiety-like and depressive-like behavior, as well as accumulating motor dysfunction. Perhaps the most consistent behavioral feature of A53T mice was a reduced level of locomotor activity which was evident on varied analyses from several different tests. This is consistent with increased clearance of DA from striatal synapses, although increased uptake of DA is not likely to be the only factor involved in the reduced activity. To provide context, it should be considered that DAT over-expressing mice have increased striatal DA clearance of a similar magnitude, yet no change in basal locomotor activity [61]. This suggests that the reduced activity level of the A53T α-Syn mice is only partially dependent on the increase in DA uptake. Furthermore, the enhancement of striatal DA uptake fades with aging, yet locomotor activity remains low, and motor strength and endurance are additionally lost. A related mouse model of A53T α-Syn over-expression with a similar reduction in locomotor activity [6] also exhibited robust changes in both pre-synaptic and post-synaptic dopaminergic function including increased striatal DA content, enhanced expression of DA receptors, and reduced expression of DA metabolizing enzymes [62]. This reflects a circumstance where DA neurotransmission is impaired, resulting in sensitization of the synapses. These changes are consistent with increased DA uptake capacity, but suggest that DA release probability, a measure of the rate of DA vesicle fusion with the synaptic membrane, may also be reduced in the presence of excess α-Syn [63]–[65]. Accumulation with aging of both β-Syn and γ-Syn, though likely to normalize DAT trafficking as proposed here, could have the additional effect of exacerbating the perturbation of DA release, and thus may simultaneously contribute to the overall lowered activity level.

The decreased immobility scores on the FST in older A53T mice are more difficult to align with what is known about the reduced dopaminergic function and motor activity in these animals. The FST primarily measures the behavioral response to entrapment, with swimming and climbing behaviors viewed as an attempt to escape, while immobility is viewed as an indication of stress-induced despair or depression [15]. It is important to note that while less active in the OFT and EPM, A53T mice were more active in the FST, with reduced immobility scores (increased swimming) at 4 and 8 months. The pattern of reduced depressive-like behavior diverged dramatically at 12 months, as symptomatic A53T mice (WHT latency<20 s) adopted an immobile posture throughout the FST period, while asymptomatic A53T animals maintained lower immobility scores compared to WT. The increase in FST immobility scores in symptomatic A53T mice suggests that increased depressive-like behaviors observed in other rodent models of PD (primarily neurotoxin models) may be linked to the acute loss of dopaminergic tissues and development of motor impairment rather than the development of pre-motor symptoms analogous to the co-morbidity of depression with PD (for review see [66]). Successfully modeling the constellation of non-motor symptoms that accompany PD, especially those that typically occur prior to clinical diagnosis, remains a pressing goal for pre-clinical researchers that could provide a means for testing prophylactic treatments or other therapeutic measures [15], [16]. While the A53T mouse lines are considered one of the more successful models of PD and synucleinopathy [3], the observed reductions in anxiety-like and depressive-like behaviors are essentially opposite what would be predicted based on clinical reports. In addition, some reports show that striatal DA concentration may actually be elevated in a similar A53T mouse model [62], [65], a finding that is clearly at odds with the accepted pathophysiology of PD [67]. Also, administration of DA or L-DOPA fails to restore normal electrophysiologal properties to striatal neurons in A53T mice [68]. Taken together with our work here, these studies suggest that A53T mice may not be ideal for modeling the full range of PD-associated symptoms, or at least that only limited inferences should be drawn from A53T mouse models with regard to the progression of sporadic PD.

In contrast, the vesicular monoamine transporter 2 deficient mouse (VMAT2 LO) undergoes a more gradual loss of monoamine storage capacity that is associated with increased depressive-like behavior in aged VMAT2 LO animals [69]. Younger VMAT2 LO mice have FST immobility scores similar to WT, suggesting that their later increase in depressive-like behavior is linked to the progressive loss of monoamine function, including NE and serotonin in addition to DA. This type of functional loss is only partially reproduced in A53T mice, as a decrease in SNpc neurons was not accompanied by a loss of striatal MSN dendritic spine density. Previously, there was limited reported evidence of nigrostriatal degeneration in aging A53T α-Syn mice [3], [43], with investigations focused primarily on severe synucleinopathy and motor neuron losses in the spinal cord [6]–[8], [70], [71]. More recently, we have reported that TH-expressing neurons in the SN of A53T α-Syn mice were reduced by 30% [24]. A similar result has been reproduced here, where a more thorough analysis found a substantial (∼20%) and statistically significant loss of both total and TH-expressing SNpc neurons in aged A53T α-Syn mice. These results argue against the prior consensus that dopaminergic pathology is absent in A53T α-Syn mice. An analysis of striatal medium spiny neurons (MSN), however, showed that dendritic spine density was unaltered as late as ten months in A53T α-Syn mice. While this presents difficulties for a clear description of the dopaminergic status of these animals, the apparent sparing of MSN spine density is not necessarily inconsistent with a partial loss of dopaminergic innervation. MSN make up 95% of striatal neurons, and are subdivided into several distinct classes that are nonetheless indistinguishable in terms of morphology or anatomical distribution [72]. Recent work has shown that in a toxin-based mouse model of parkinsonism the MSN sub-type expressing the D2 dopamine receptor undergoes selective dendritic spine loss, while neighboring MSN expressing the D1 dopamine receptor are largely unaffected [73]. Similar works showing substantial loss of MSN dendritic spine density typically involve lesions causing SNpc neuron loss much greater than 20% [72], and so it is possible that the limited loss of TH-expressing cells in the present study is insufficient to produce significant dendritic spine remodeling. Furthermore, MSN sub-types were not differentiated here; future work should examine both D1 and D2 dopamine receptor expressing cells to determine whether these functionally distinct populations are differentially affected in the A53T α-Syn mouse. Further analysis of striatal function seems especially needed given the evidence presented here and elsewhere [24] that these animals undergo SNpc cell losses. Furthermore, we have shown that the biochemical phenotype of A53T α-Syn mice includes excess cytosolic DA exposure, and the time course of these changes parallel the behavioral phenotype of these mice. We propose that the function of increased Tau phosphorylation in this context is more complex, being a marker of DA-induced stress kinase activation in the initial stages that fades as DAT modulation by β-Syn and γ-Syn is restored. These findings support our view of the central importance of a linkage between Tau hyper-phosphorylation and the development of synucleinopathy in dopaminergic tissues, including the progression of PD [24], [25], [27]–[29], [50], [74].

## Supporting Information

Figure S1 **Motor activity.** OFT, EPM, and rotarod tests were performed on WT and A53T mice at 2-12 months of age (n = 14–18 per group) to analyze motor activity and function. (A) Total line breaks (transition between any two regions) on the OFT and (B) total distance traveled on the EPM were measured over a ten min period by automated video tracking using ANY-maze software. Results are presented as mean ± SEM and were analyzed by two-way ANOVA with Bonferroni post-hoc tests comparing each A53T group to age-matched controls (***p<0.001). (C) Linear regression shows rate of decline in rotarod latency to fall (WT = -9.6±0.9 s/month; A53T = −7.6±2.8 s/month). Slopes were analyzed by t-test comparing WT to A53T (no significant difference detected).
(TIF)

Figure S2 **Anxiety-like behavior.** Anxiety-like behavior was analyzed by the OFT and EPM on WT and A53T mice at 2-12 months of age (n  =  14-18 per group). (A) Total center zone entries on the OFT and (B) total open arm entries on the EPM were measured by automated video tracking using ANY-maze software. Results are presented as mean ± SEM and were analyzed by two-way ANOVA with Bonferroni post-hoc tests comparing each A53T group to age-matched controls (*p<0.05; ***p<0.001).
(TIF)

Figure S3 **NET distribution and function.** Expression and distribution of NET in the hippocampus was analyzed by immunoblot on protein from (A) total lysates (TL) or synaptosomal plasma membrane fractions (SPM). Actin or cadherin expression, respectively, were analyzed as loading controls. Representative blot images from each genotype at each age are presented. Band optical density (OD) of (B) TL and (C) SPM NET relative to loading controls is presented as percent of two month old WT (mean ± SEM) and was analyzed by two-way ANOVA with Bonferroni post-hoc tests comparing each A53T group to age-matched controls. (D) Uptake of [^3^H]-NE into striatal synaptosomes isolated from WT and A53T mice at 2-12 months of age was measured in triplicate from six animals per group and is presented as percent of age-matched WT control (mean ± SEM). Non-specific uptake was determined in the presence of 1 µM desipramine HCl and has been subtracted. Comparisons between WT and A53T α-Syn at each age were made by t-test (*p<0.05).
(TIF)

Figure S4 **Synuclein accumulation and aggregation in the hippocampus.** (A) Expression of Syn proteins was analyzed by immunoblot on hippocampal total lysates with actin expression analyzed as a loading control. (B) Band optical density (OD) relative to actin is presented as percent of two month old A53T mice (A53T human α-Syn) or two month old WT (total α-Syn and endogenous Syn proteins) and was analyzed by two-way ANOVA with Bonferroni post-hoc tests comparing two and 12 month old A53T (no significant differences detected). (C) Solubility of α-Syn was analyzed by immunoblot on hippocampal homogenate extracted with 1% TX-100 and centrifuged at 15,000 RCF for 60 min. Insoluble pellets were further extracted with the addition of 2% SDS and fractions were analyzed in parallel by immunoblot. Representative blot images from each genotype at each age are presented with approximate molecular mass of nearest protein ladder bands indicated (M_r_).
(TIF)

Figure S5 **Hippocampal accumulation of PHF-1 Tau and Tau kinase activation.** (A) Phosphorylation of Tau protein at the PHF-1 epitope, expression of Tau kinases, and phosphorylation of kinases at activating sites was analyzed by immunoblot on hippocampal total lysates. (B) Band optical density (OD) relative to appropriate loading controls is presented as percent of two month old WT (mean ± SEM) and was analyzed by two-way ANOVA with Bonferroni post-hoc tests comparing each A53T group to age-matched controls (no significant differences detected).
(TIF)
