# Supplementary figures and images for: Age-Dependent Effects of A53T Alpha-Synuclein on Behavior and Dopaminergic Function
Source: PLoS One. 2013 Apr 1;8(4):e60378. doi: 10.1371/journal.pone.0060378 (PMC3613356; doi:10.1371/journal.pone.0060378)

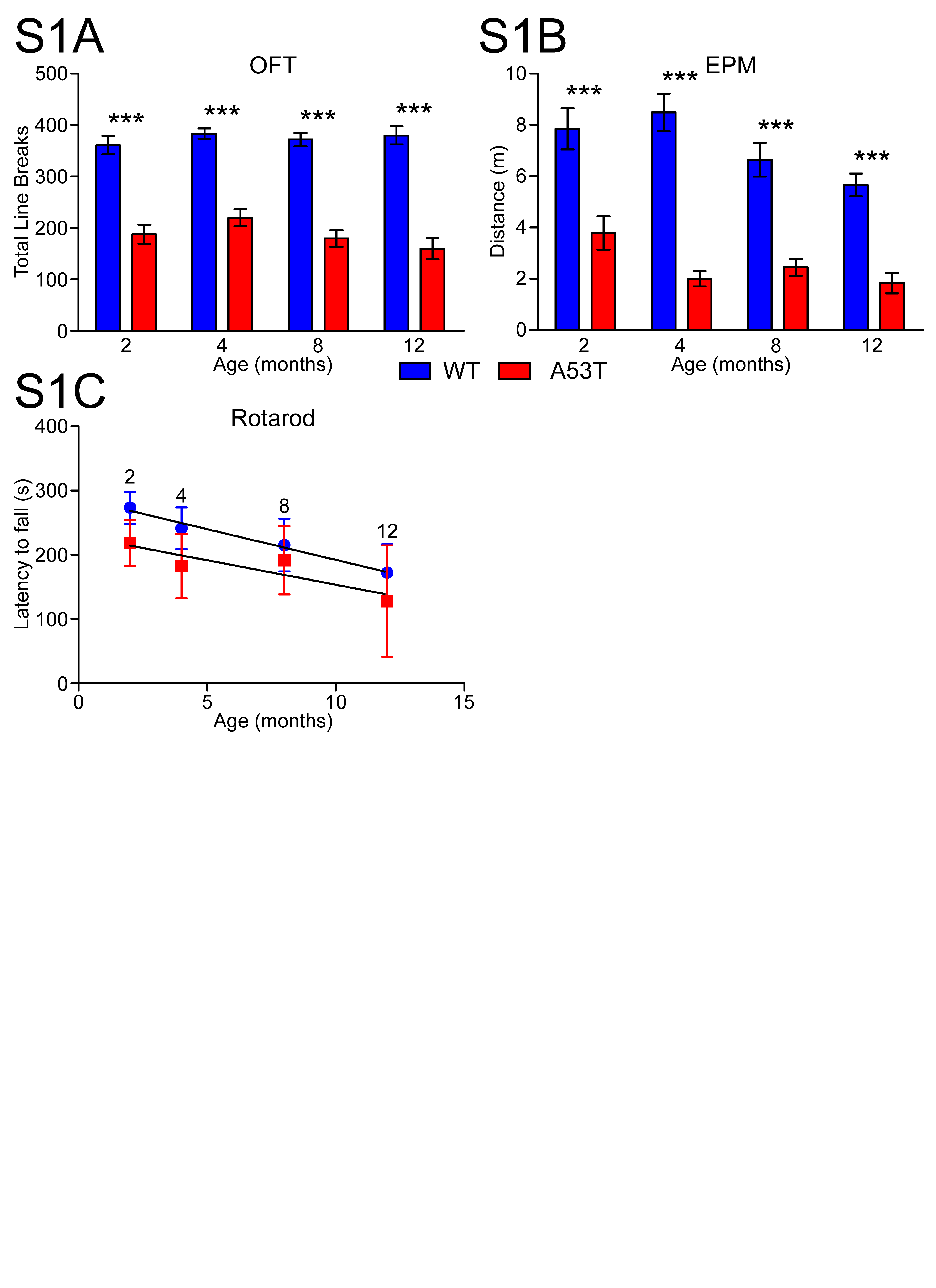

Supplement: Figure S1 — Motor activity. OFT, EPM, and rotarod tests were performed on WT and A53T mice at 2-12 months of age (n = 14–18 per group) to analyze motor activity and function. (A) Total line breaks (transition between any two regions) on the OFT and (B) total distance traveled on the EPM were measured over a ten min period by automated video tracking using ANY-maze software. Results are presented as mean ± SEM and were analyzed by two-way ANOVA with Bonferroni post-hoc tests comparing each A53T group to age-matched controls (***p<0.001). (C) Linear regression shows rate of decline in rotarod latency to fall (WT = -9.6±0.9 s/month; A53T = −7.6±2.8 s/month). Slopes were analyzed by t-test comparing WT to A53T (no significant difference detected). (TIF) [file pone.0060378.s001.tif]

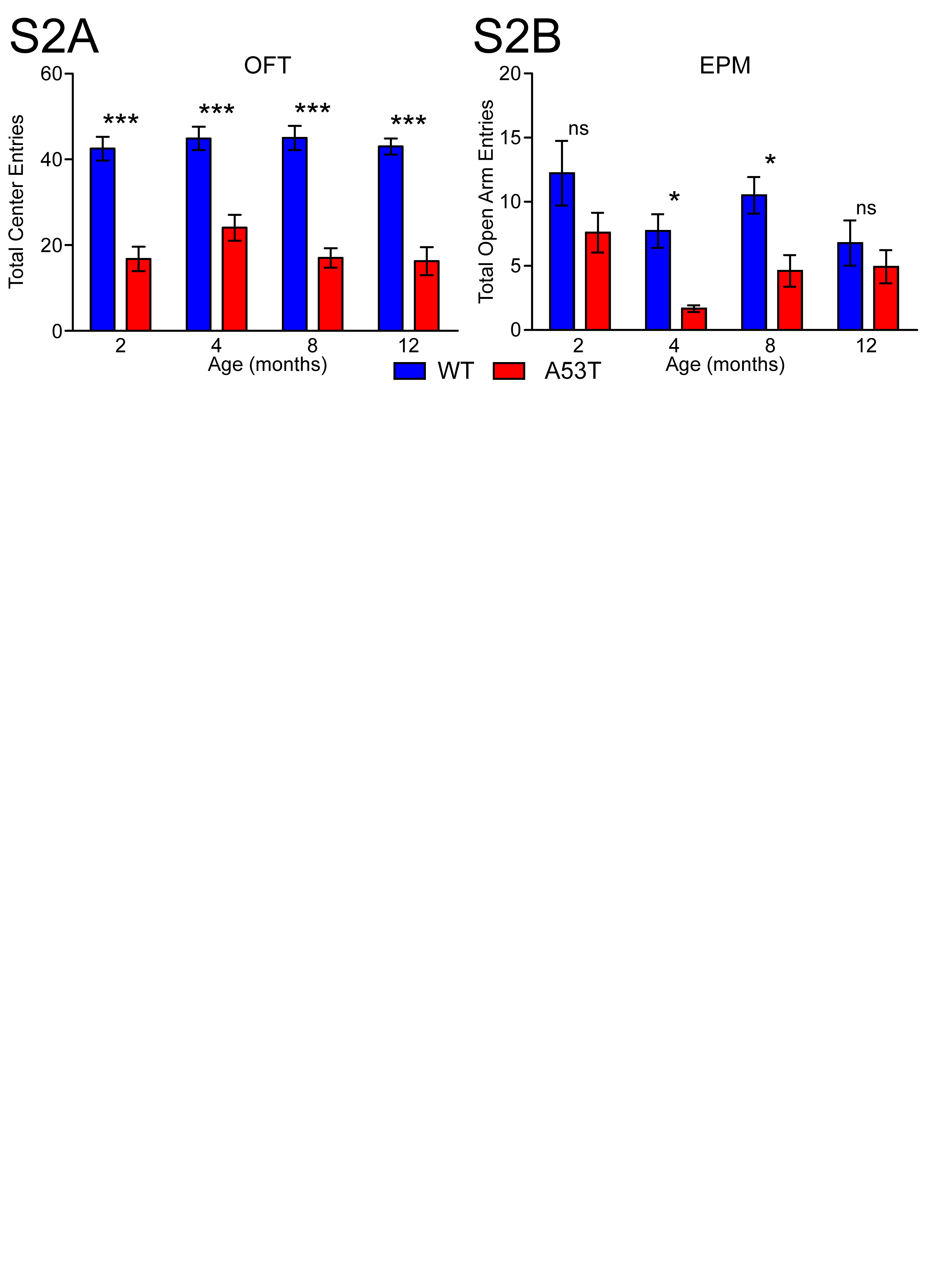

Supplement: Figure S2 — Anxiety-like behavior. Anxiety-like behavior was analyzed by the OFT and EPM on WT and A53T mice at 2-12 months of age (n = 14-18 per group). (A) Total center zone entries on the OFT and (B) total open arm entries on the EPM were measured by automated video tracking using ANY-maze software. Results are presented as mean ± SEM and were analyzed by two-way ANOVA with Bonferroni post-hoc tests comparing each A53T group to age-matched controls (*p<0.05; ***p<0.001). (TIF) [file pone.0060378.s002.tif]

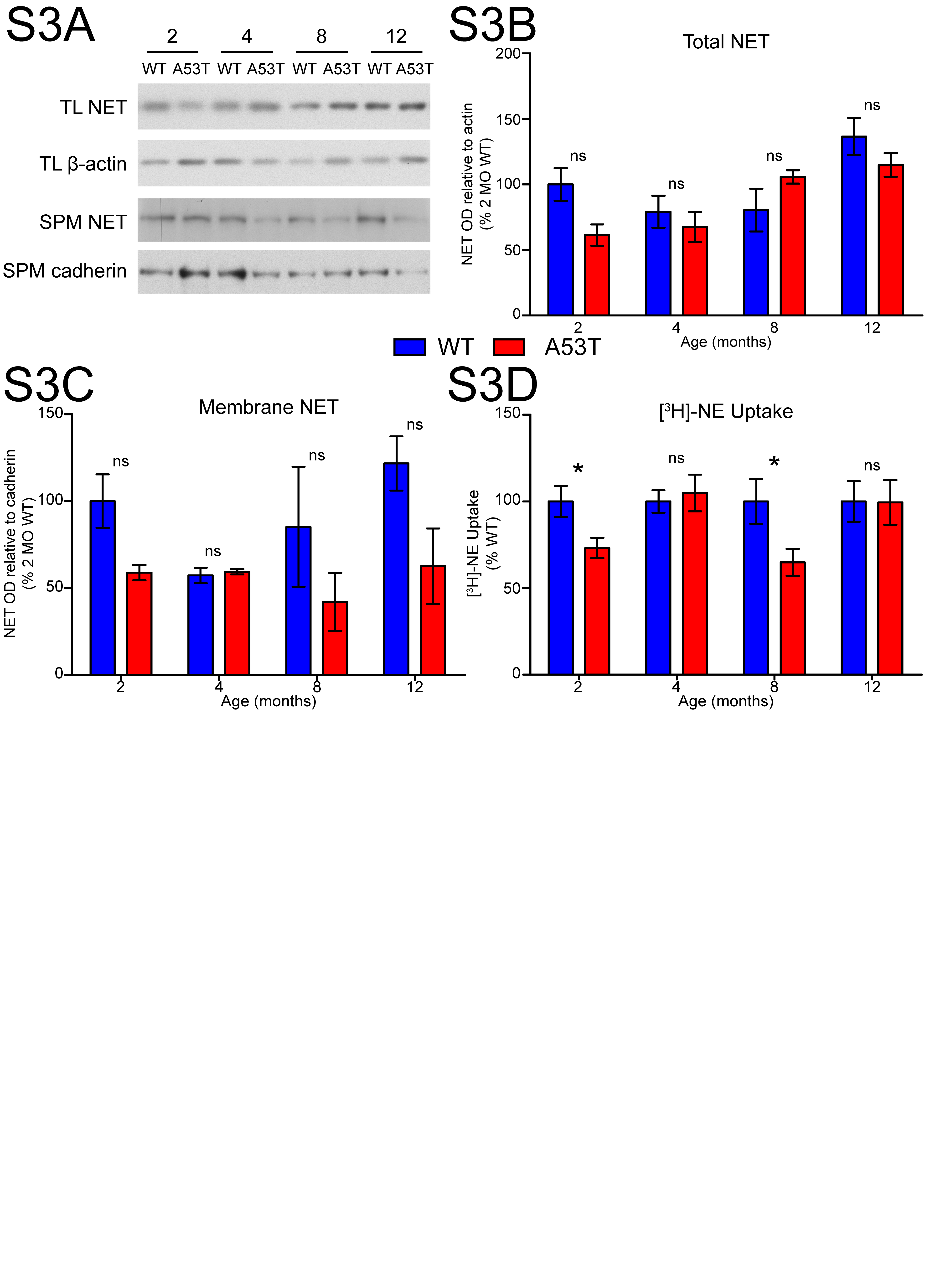

Supplement: Figure S3 — NET distribution and function. Expression and distribution of NET in the hippocampus was analyzed by immunoblot on protein from (A) total lysates (TL) or synaptosomal plasma membrane fractions (SPM). Actin or cadherin expression, respectively, were analyzed as loading controls. Representative blot images from each genotype at each age are presented. Band optical density (OD) of (B) TL and (C) SPM NET relative to loading controls is presented as percent of two month old WT (mean ± SEM) and was analyzed by two-way ANOVA with Bonferroni post-hoc tests comparing each A53T group to age-matched controls. (D) Uptake of [3H]-NE into striatal synaptosomes isolated from WT and A53T mice at 2-12 months of age was measured in triplicate from six animals per group and is presented as percent of age-matched WT control (mean ± SEM). Non-specific uptake was determined in the presence of 1 µM desipramine HCl and has been subtracted. Comparisons between WT and A53T α-Syn at each age were made by t-test (*p<0.05). (TIF) [file pone.0060378.s003.tif]

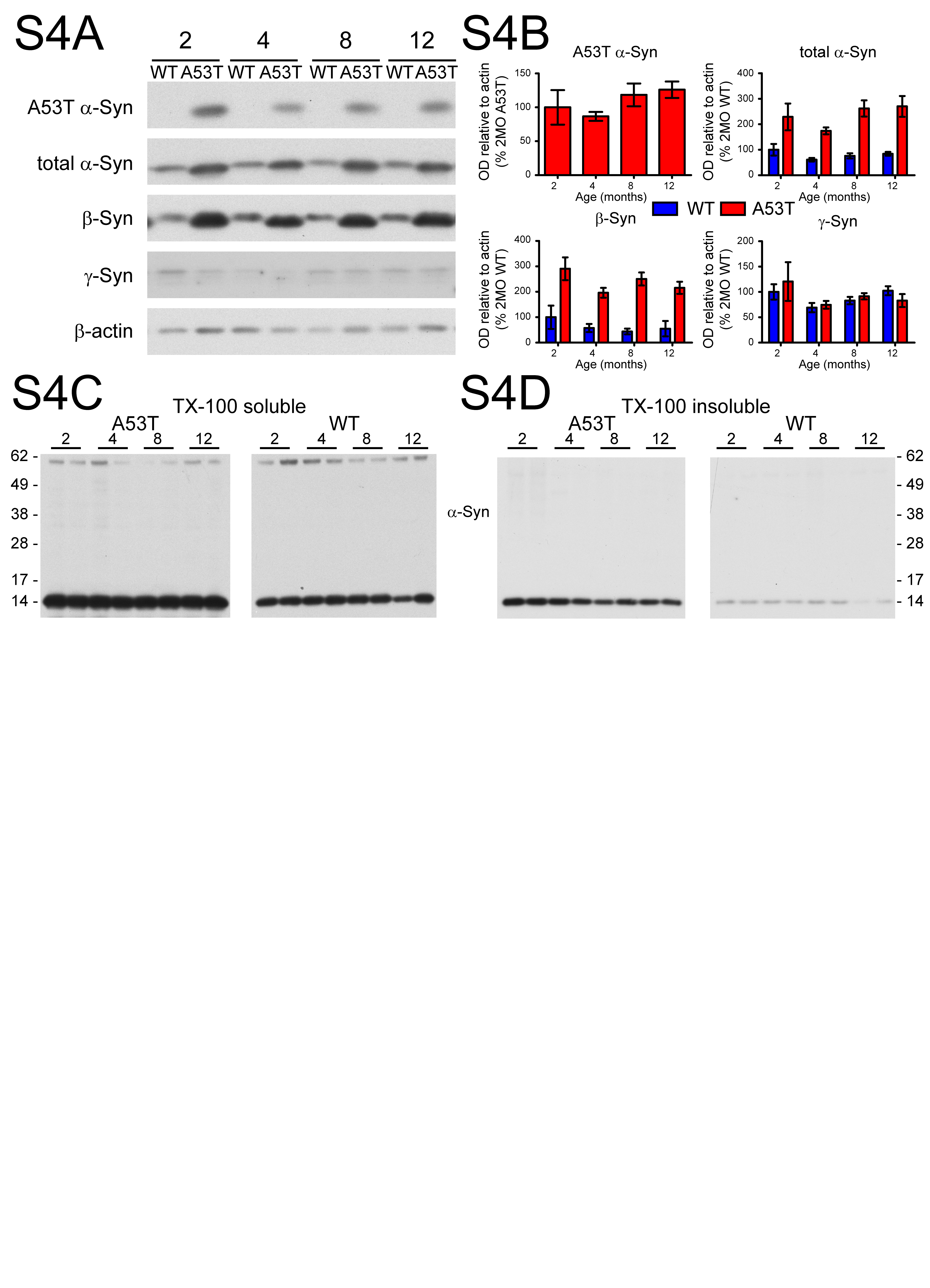

Supplement: Figure S4 — Synuclein accumulation and aggregation in the hippocampus. (A) Expression of Syn proteins was analyzed by immunoblot on hippocampal total lysates with actin expression analyzed as a loading control. (B) Band optical density (OD) relative to actin is presented as percent of two month old A53T mice (A53T human α-Syn) or two month old WT (total α-Syn and endogenous Syn proteins) and was analyzed by two-way ANOVA with Bonferroni post-hoc tests comparing two and 12 month old A53T (no significant differences detected). (C) Solubility of α-Syn was analyzed by immunoblot on hippocampal homogenate extracted with 1% TX-100 and centrifuged at 15,000 RCF for 60 min. Insoluble pellets were further extracted with the addition of 2% SDS and fractions were analyzed in parallel by immunoblot. Representative blot images from each genotype at each age are presented with approximate molecular mass of nearest protein ladder bands indicated (Mr). (TIF) [file pone.0060378.s004.tif]

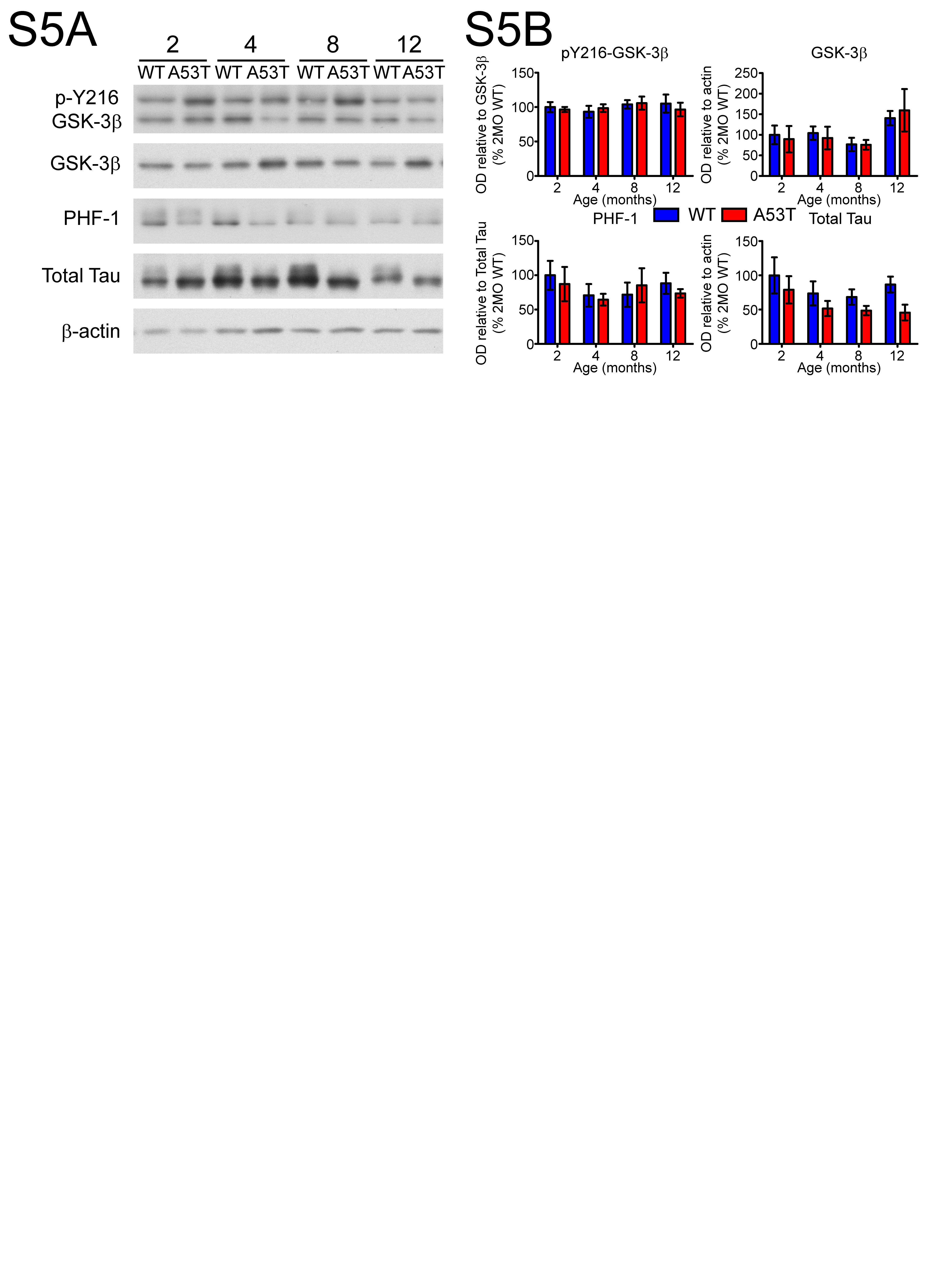

Supplement: Figure S5 — Hippocampal accumulation of PHF-1 Tau and Tau kinase activation. (A) Phosphorylation of Tau protein at the PHF-1 epitope, expression of Tau kinases, and phosphorylation of kinases at activating sites was analyzed by immunoblot on hippocampal total lysates. (B) Band optical density (OD) relative to appropriate loading controls is presented as percent of two month old WT (mean ± SEM) and was analyzed by two-way ANOVA with Bonferroni post-hoc tests comparing each A53T group to age-matched controls (no significant differences detected). (TIF) [file pone.0060378.s005.tif]
